# pH-Dependent Photophysical Properties of Metallic Phase MoSe_2_ Quantum Dots

**DOI:** 10.3390/ma15144945

**Published:** 2022-07-15

**Authors:** Boemjin Ko, Jaegyu Ahn, Sung Ho Song

**Affiliations:** Division of Advanced Materials Engineering, Kongju National University, Cheonan 330-717, Chungcheongnam-do, Korea; qjawls5004@naver.com (B.K.); jaegyuan@naver.com (J.A.)

**Keywords:** MoSe_2_, quantum dots, photophysics, pH effect, size effect, TRPL, PLE

## Abstract

Fluorescence properties of quantum dots (QDs) are critically affected by their redox states, which is important for practical applications. In this study, we investigated the optical properties of MoSe_2_-metallic phase quantum-dots (MoSe_2_-*m*QDs) depending on the pH variation, in which the MoSe_2_-*m*QDs were dispersed in water with two sizes (Φ~3 nm and 12 nm). The larger MoSe_2_-*m*QDs exhibited a large red-shift and broadening of photoluminescence (PL) peak with a constant UV absorption spectra as varying the pH, while the smaller ones showed a small red-shift and peak broadening, but discrete absorption bands in the acidic solution. The excitation wavelength-dependent photoluminescence shows that the PL properties of smaller MoSe_2_-*m*QDs are more sensitive to the pH change compared to those of larger ones. From the time-resolved PL spectroscopy, the excitons dominantly decaying with an energy of ~3 eV in pH 2 clearly show the shift of PL peak to the lower energy (~2.6 eV) as the pH increases to 7 and 11 in the smaller MoSe_2_-*m*QDs. On the other hand, in the larger MoSe_2_-*m*QDs, the exciton decay is less sensitive to the redox states compared to those of the smaller ones. This result shows that the pH variation is more critical to the change of photophysical properties than the size effect in MoSe_2_-*m*QDs.

## 1. Introduction

Two-dimensional (2D) layered transition metal dichalcogenides (TMDs) have been extensively studied [1,2,3,4,5] and their quantum dots (*m*QDs) have attracted interest because of their potential applications [6,7,8,9]. In the TMDs structures, there are two different phases, semiconducting and metallic (1T/1T’), exhibiting miscellaneous changes in electronic and optical properties [5,7,8,9,10,11,12]. In general, semiconducting TMDs-QDs show a broadening of excitonic absorption peaks and a higher quantum yield compared to flakes [7,8,9], while the metallic phase TMDs-QDs (TMDs-*m*QDs) have features of high charge transfer efficiency and photocatalytic and electrocatalytic capabilities [9,13]. Recently, the photophysical properties of two-sized TMDs-*m*QDs were demonstrated to have different charge excitation and decay pathways, which was ascribed to defect states and valence band splitting [14]. Also, the optical energy band structure of the TMDs-*m*QDs was reported to be predominantly affected by edge oxidation [15]. Because the defect and the edge oxidation of atomically thin TMDs-*m*QDs strongly depends on the environmental conditions [16,17,18,19], to study the photophysical properties as changing the pH of solution and the size relating to defects is essential for the practical applications.

**Figure 1:**
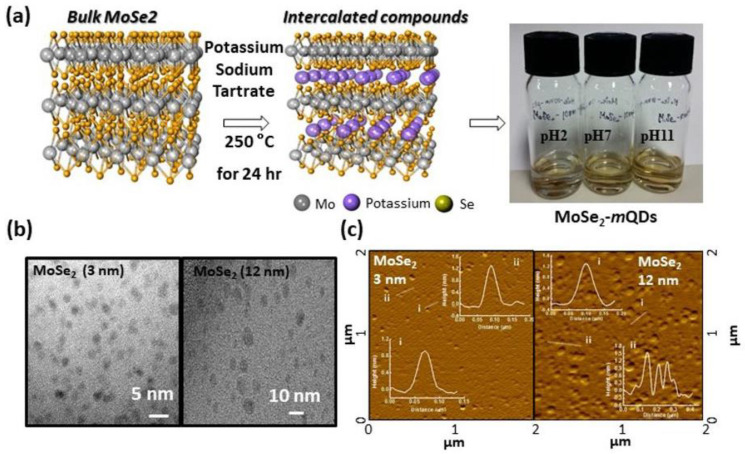
Synthesis and characterization of MoSe_2_-*m*QD [15]. (**a**). Schematic illustration of synthetic process of MoSe_2_-*m*QD and digital images of dispersed MoSe_2_-*m*QD in water with ph 2, 7, and 11. (**b**). TEM images of MoSe_2_-*m*QD-*L* (right) and MoSe_2_-*m*QD-*S* (left). (**c**). AFM images of MoSe_2_-*m*QD-*S* (left) and MoSe_2_-*m*QD-*L* (right) with height profiles.

Studies of the effects of redox on TMDs flakes have largely been for tuning optical and physical properties, however, the pH dependence of TMDs’ PL is still suggestive and speculative based on the results measured from TMDs flakes. So far, the pH-dependent optical properties of TMDs-*m*QDs have been rarely studied compared to those of semiconducting TMDs-QDs [9,20,21,22]. This is because the 1T/1T’ metallic phase of TMDs is known to be less stable than the 2H semiconducting phase, in addition to having a large band gap and high surface to volume ratios. Semiconducting TMDs-QDs have been reported to exhibit different quantum yields with varying emission wavelengths depending on their size, surrounding conditions, and edge functionalization [4,9,23,24,25,26,27]. Mishra et al. showed that semiconducting MoS_2_-QDs had a pH-dependent fluorescence switching behavior, which was mainly ascribed to the surface-absorbed functional groups [26]. For tin disulfide quantum dots, the quantum yield (QY) was higher in pH~1 (QY~5.32%) compared to that (QY~1.17%) in pH~12, and it was proposed that this effect was due to the protonation and deprotonation of edge functional groups. Several research groups including ours have developed a synthesis process for TMDs-(*m*)QDs and studied their optical properties (Figure 1a, see details in [5,10]) [5,14]. In the process, the TMDs were co-intercalated alkali metal-organic compounds and exfoliated to be *m*QDs showing an exotic exciton dynamics [14,25,28]. The PL shift in TMDs-(*m*)QDs is thought to be due to the size-dependent quantum confinement effect and the localized (delocalized) states theoretically related to the vacancy and oxidation defects. The as-prepared MoS_2_-*m*QDs were also examined for bio-imaging applications and showed biocompatibility with bright luminescence [29]. However, TMDs-*m*QDs have usually been studied in the neutral state, how environmental influences such as acidic or alkaline solution combined with the size effect on the TMDs-*m*QDs has not been thoroughly probed yet.

In the present work, we investigate the effect of redox states on the photophysical properties of two sizes (Φ~3 nm and ~12 nm) of MoSe_2_-*m*QDs. The separately collected two sizes MoSe_2_-*m*QDs were re-dispersed in water with three different pH of ~2, 7, and 11; and their photoluminescence (PL), UV-absorbance, excitation wavelength-dependent PL (PLE), and time-resolved PL (TRPL) were measured. For the PLE, the excitation wavelength (λ_ex_) changed from 250 nm to 550 nm, showing that the emission wavelengths red-shifted as the λ_ex_ increased. From the TRPL spectra of smaller MoSe_2_-*m*QDs depending on pH, the exciton radiatively decayed with a weak red-shift of peak position in pH 2, while it increased the red-shift as the pH increased. On the other hand, the larger ones did not clearly show the red-shift of PL peak as varying the pH.

## 2. Materials and Methods

### 2.1. Materials

Bulk MoSe_2_ was purchased from 4 sicence (iNexus, Inc., Seoul, Korea). Potassium sodium tartrate was purchase from Sigma-Aldrich (St. Louis, MO, USA) and used without further purification. Chemicals including NaOH, HCl, ethanol, methanol, and acetone were purchase from Chemicals Duksan corp. and used without further purification.

### 2.2. Preparation of MoSe_2_-mQDs and Dispersion

Potassium sodium tartrate was selected in order to synthesize metallic phase MoSe_2_-*m*QDs at low temperature to minimal damage [5,14,29]. The detail for fabrication can be consulted in [14]. Briefly, the initial was the mixing and grinding of potassium sodium tartrate (200 mg) with MoSe_2_ (20 mg). Then, the ground homogeneous mixtures reacted with the autoclave vessel at 250 °C for 12 h then instantly exfoliated in water with sonication. The sizes of the MoSe_2_-*m*QDs were controlled by AAO (20 nm) filtration following the filtration (10,000 and 8000 NMWL, Amicon Ultra-15) methods, and then dialyzed in dialysis tubing, which simultaneously removed the remaining salts. Finally, the MoSe_2_-*m*QDs were obtained as dispersed solution in water. After being dried, the MoSe_2_-*m*QDs were re-dispersed in 10 mL of water with pH 2, 7, and 11.

### 2.3. Characterization

The morphology of MoSe_2_-*m*QDs was analyzed using an atomic force microscope (AFM, SPA400, SⅡ, Chiba, Japan) in tapping mode under ambient conditions. UV-Vis spectra (Shimadzu UV-3101PC spectrometer, EVISA, Switzerland), fluorescence spectra (Perkin-Elmer LS 55 luminescence spectrometer, Waltham, MA, USA), and transmission electron microscopy (TEM, Tiatan cubed G2 60-300, FEI, Hillsboro, OR, USA) analyses were conducted. TEM samples were prepared by drying a droplet of the MoSe_2_-*m*QDs suspensions on a carbon grid. The photoluminescence (PL) measurements were carried out using a 325 nm He-Cd continues-wave (CW) laser, a monochromatic light from a 300 W-xenon lamp, and UV spectrometers (Maya2000, Ocean Optics, Dunedin, FL, USA) as a PL detector at room temperature. The PL excitations were measured by monochromatic light from a 300 W Xenon lamp and a highly sensitive photomultiplier tube as a PL detector. In order to elucidate the recombination dynamics, we carried out time-resolved PL experiments. A mode-locked femto-second pulsed Ti: sapphire laser (Coherent, Chameleon Ultra II, Santa Clara, CA, USA) system was used as an excitation source, and the five wavelengths of the pulsed Ti:sapphire laser (266 nm, 300 nm, 350 nm, 400 nm, and 450 nm) were employed. A streak camera (Hamamatsu, Japan, C7700-01) was utilized to measure the decay profile of the PL spectra at room temperature.

## 3. Results

### 3.1. Preparation and Structural Characterization

To study the photophysical properties of TMDs-*m*QDs depending on their redox states, we synthesized and separately collected two different sizes of MoSe_2_-*m*QDs (Φ~3 nm, MoSe_2_-*m*QDs-*S* and 12 nm, MoSe_2_-*m*QDs-*L*) and re-dispersed them in the water with pH 2, 7, and 11 (Figure 1a). Figure 1b shows transmission electron microscope (TEM) images of MoSe_2_-*m*QDs with average sizes of ~3 nm and ~12 nm. From the AFM images (Figure 1c), the height of the quantum dots is 1~2 nm, corresponding to a thickness of less than three layers of MoSe_2_. The detailed structural properties of the MoSe_2_-*m*QDs were reported in [14].

### 3.2. Photoluminescence (PL) and UV Absorbance

**Figure 2:**
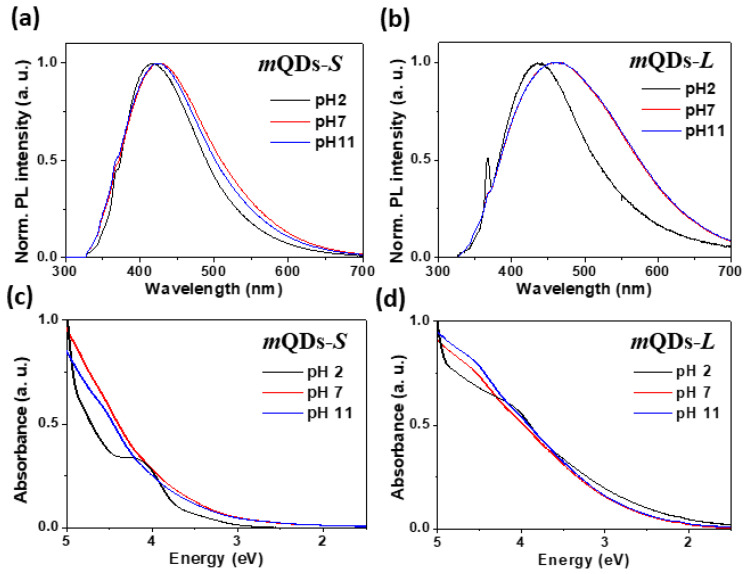
Photoluminescence (PL) spectra and UV-vis absorbance. (**a**,**b**). PL spectra of MoSe_2_-*m*QD-*S* (**a**) and MoSe_2_-*m*QD-*L* (**b**) measured with an excitation wavelength of 325 nm. (**c**,**d**). UV absorbance of MoSe_2_-*m*QD-*S* (left) and MoSe_2_-*m*QD-*L* (right).

Figure 2a,b shows the PL spectra of MoSe_2_-*m*QDs-*S* and MoSe_2_-*m*QDs-*L,* respectively, measured with an excitation wavelength (λ_ex_) of 325 nm. For the MoSe_2_-*m*QDs-*S* (Figure 2a), the PL peak positioned at 418 nm with a full width at half maximum (FWHM) of 0.79 eV in pH 2, which is slightly red-shifted (Δλ~7 nm) and enlarged (ΔFWHM~0.07–8 eV) at pH 7 and 11. On the other hand, the PL peak of MoSe_2_-*m*QDs-*L* appears at 437 nm with a FWHM of 0.84 eV, which further red-shifts to 461 nm (Δλ~24 nm) and broadens to 1.07 and 1.08 eV of FWHM (ΔFWHM~0.23, 0.24 eV) in pH 7 and 11 (Figure 2b). By comparing the PL spectra of MoSe_2_-*m*QDs-*S* and MoSe_2_-*m*QDs-*L,* the pH variation was determined to have more effectively changed the PL spectrum than the size effect. Figure 2c,d shows the UV-vis absorbance spectra of MoSe_2_-*m*QDs-*S* and MoSe_2_-*m*QDs-*L*, respectively, according to the variation in pH. The absorbance spectrum of MoSe_2_-*m*QDs-*S* in pH 2 appears to have discrete absorption energy bands, however, this dimmed in pH 7 and 11. On the other hand, the MoSe_2_-*m*QDs-*L* shows a monotonically increasing absorbance at excitation energies lower than 4 eV, which is a feature of the metallic state QDs [14]. At excitation energies higher than 4 eV, the slope of absorbance similarly changed in both of MoSe_2_-*m*QDs. In addition, the absorbance of MoSe_2_-*m*QDs-*L* started to increase at a lower excitation energy than that of MoSe_2_-*m*QDs-*S*, regardless of pH. This analysis implies that the protonation besides the size effect enforces the quantum confinement in the MoSe_2_-*m*QDs, which is obviously observed in the MoSe_2_-*m*QDs-*S* with a size close to the Bohr exciton diameter [30]. The quantum yields (QY) of MoSe_2_-*m*QDs-*S* were 3.7%, 5.2%, and 5% at pH 2, 7, and 11, respectively, as measured using an absolute PLQY measurement system with an excitation wavelength of 325 nm. The PLQY could not be estimated in MoSe_2_-*m*QDs-*L* due to the low quantum efficiency. The values of peak position, FWHM, and QYs are listed on Table 1. These results imply that the oxidation of MoSe_2_-*m*QDs relieves the localization of the excited charges and the quantum confinement effect. On the other hand, the protonation of MoSe_2_-*m*QDs induces the energy band structure to be more discrete, which might be a cause of the low quantum efficiency, suggesting that the partial oxidation enhances the quantum efficiency of MoSe_2_-*m*QDs through forming sub-bands, which assists the intraband transition of charges.

### 3.3. Excitation Wavelength Dependent PL (PLE)

**Figure 3:**
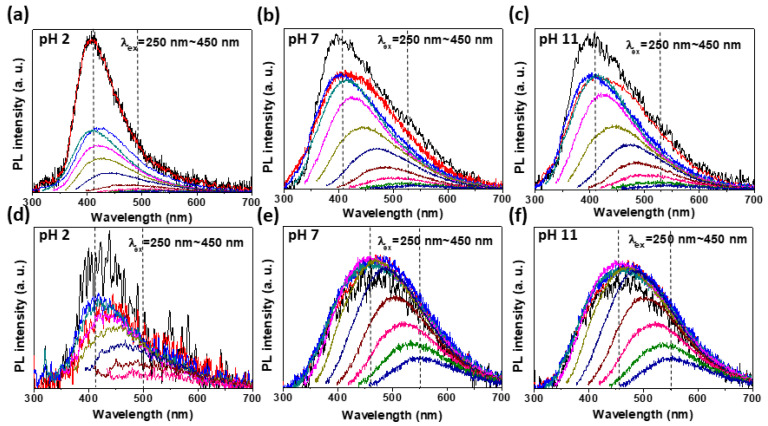
Excitation wavelength-dependent Photoluminescence (PLE) spectra. (**a**–**c**). PLE spectra of MoSe_2_-*m*QD-*S* excited by λ*_Ex_*~250 (black), 270 (red), 290 (blue), 310 (dark cyan), 330 (Magenta), 350 (dark yellow), 370 (navy), 390 (wine), 410 (pink), 430 (olive), 450 (royal) nm, respectively, at pH 2 (**a**), pH 7 (**b**), and pH 11 (**c**). The gap between excitation wavelengths is 20 nm. (**d**–**f**). PLE spectra of MoSe_2_-*m*QD-*L* excited by λ*_Ex_*~250–470 nm at pH 2 (**d**), pH 7 (**e**), and pH 11 (**f**). The gap between excitation wavelengths is 20 nm.

To examine the variation in optical energy band structure depending on pH, we measured the excitation wavelength (λ_ex_)-dependent PL (PLE) from MoSe_2_-*m*QDs in pH 2, 7, and 11. Figure 3 is the PLE spectra of MoSe_2_-*m*QDs-*S* (a, b, and c) and MoSe_2_-*m*QDs-*L* (d, e, and f) as a function of λ_ex_. Similar to WS_2_, MoS_2_, and pristine MoSe_2_-GQs [14,25,28], the MoSe_2_-*m*QDs showed a red-shift in the PLE peak as the λ_ex_ increased for all pH values. The PLE spectra of MoSe_2_-*m*QDs-*S* in pH 2 (Figure 3a) exhibited a peak shift from ~410 nm to ~490 nm (Δλ~80 nm) as the λ_ex_ varied from 250 nm to 450 nm. When the pH increased to 7 and 11, the peak shift went up to 535 nm (Δλ~125 nm) at λ_ex_~450 nm, which is a 45 nm further red-shift compared to that in pH 2. This phenomena has been similarly observed in WS_2_-QDs due the size effect, and in MoS_2_-QDs due to the pH effect [25,26]. However, it is important to note that the PLE peak intensity of MoSe_2_-*m*QDs-*S* sharply decreased in the range of λ_ex_ larger than ~310 nm in pH 2, while it slowly decreased with increasing λ_ex_ in pH 7 and 11. This indicates that in MoSe_2_-*m*QDs-*S* the pH variation does critically affect the decay pathway of excitons, most likely due to the formation of sub-bands related to the redox state of MoSe_2_-*m*QDs. The oxidation can induce an extension of band-edge, resulting in some excited charges transiting to the lower energy state. This is in accordance with the analysis of results shown in Figure 2 and Table 1. A peak shift in the PLE spectra was similarly observed in MoSe_2_-*m*QDs-*L* (Figure 3d–f). In pH 2, the PLE peak positions of MoSe_2_-*m*QDs-*L* depending on the λ_ex_ were similar to those of MoSe_2_-*m*QDs-*S* at pH 2, supporting the idea that the protonation of MoSe_2_-*m*QDs enforces the exciton decay at the intrinsic energy states. However, in pH 7 and 11, the PLE peaks at λ_ex_~250–310 nm appeared at ~460 nm, which are red-shifted as much as ~50 nm compared to those in pH 2. The PLE peak position at the λ_ex_~450 nm was ~550 nm (Δλ~90 nm) compared to that at λ_ex_~250 nm, which is a smaller peak shift compared to that (Δλ~125 nm) shown in MoSe_2_-*m*QDs-*S*. This means that the effect of pH variation in MoSe_2_-*m*QDs-*L* is weaker than that in MoSe_2_-*m*QDs-*S*, although it is stronger than the size effect.

### 3.4. Time Resolved PL (TRPL)

**Figure 4:**
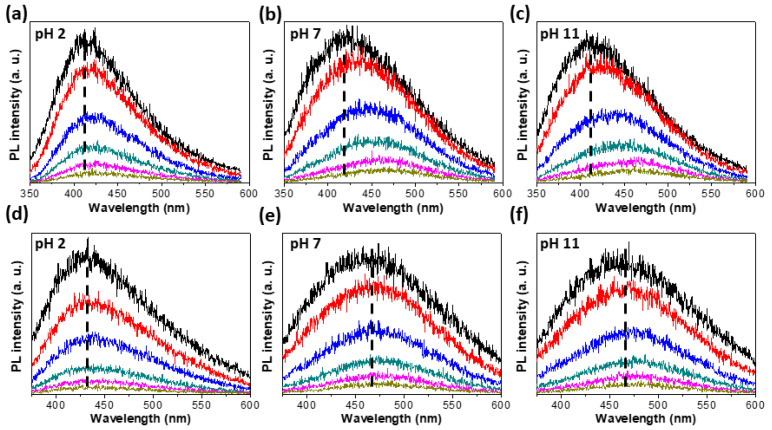
Time-resolved photoluminescence (TRPL). (**a**–**c**). TRPL spectra measured from MoSe_2_-*m*QD-*S* at pH 2 (**a**), pH 7 (**b**), and pH 11 (**c**) with λ*_ex_*~266 nm. (**d**–**f**). TRPL spectra measured from MoSe_2_-*m*QD-*L* at pH 2 (**d**), pH 7 (**e**), and pH 11 (**f**) with λ*_ex_*~310 nm. Each PL spectrum has the intensity accumulated for 0–2 (black), 2–6 (red), 6–12 (blue), 12–20 (green), 20–30 (magenta), and 30–42 (dark yellow) ns.

Figure 4 is the time resolved PL (TRPL) spectra depending on the pH of MoSe_2_-*m*QDs-*S* (Figure 4a–c) and MoSe_2_-*m*QDs-*L* (Figure 4d–f) measured at λ_ex_~266 and 310 nm, respectively, from 0 ns to 42 ns with six steps of time interval. The cumulative spectra of MoSe_2_-*m*QDs-*S* for the PL measurement time interval showed a similar trend in peak shift at the three pHs as time went on (Figure 4a–c). However, the peak shift was much clearer in pH 7 and 11, from ~415 nm to ~465 nm, indicating that the charge delocalization and migration to the lower energy states easily occur when the MoSe_2_-*m*QDs are oxidized. This is in line with the analysis in Figure 3 that the decay pathway of excitons has an intrinsic bandgap in the protonated MoSe_2_-*m*QDs-*S,* while the band edge changes as the pH increases. In comparison, the MoSe_2_-*m*QDs-*L* does not show a distinct peak shift in all pHs, although the PL peak position in pH 2 is ~430 nm and shifts to ~465 nm in pH 7 and 11. Because the PL peaks of MoSe_2_-*m*QDs-*L* in pH 2 are closer to the values (~415 nm) of MoSe_2_-*m*QDs-*S* than the values (~465 nm) in pH 7 and 11, the size effect looks to be weaker than the pH effect. This again confirms that the protonation of MoSe_2_-*m*QDs enhances the exciton decay at the band states with an intrinsic bandgap.

## 4. Discussion

This result we analysed above implies that in low pH the protonation of MoSe_2_-*m*QDs enhances the quantum confinement via the localization of the energy band, while the high pH induces the extension of the band edge, resulting in the large PL peak shift. The high pH of the solution can be attributed to the oxidation of MoSe_2_-*m*QDs, which makes a difference in electron density on MoSe_2_-*m*QDs in pH 2 and pH 11. Moreover, the higher electro negativity can block the interaction of MoSe_2_-*m*QDs with water molecules, causing the PL quenching. The analysis of PLE and TRPL results indicates that the variation of pH is more effective on the change of PL properties in MoSe_2_-*m*QDs than the size effect. On the other hand, as the size of MoSe_2_-*m*QDs increases to be larger than the exciton Bohr diameter, the extrinsic band structures related to the structural and oxidation defects become the dominant decay pathway of excitons.

## 5. Conclusions

We synthesized two different sized metallic phase MoSe_2_-QDs and investigated their photophysical properties depending on pH. The MoSe_2_-*m*QDs-*S* showed narrow PL spectra in all pH compared to MoSe_2_-*m*QDs-*L* and a higher quantum yield in pH 7 and 11 than that in pH 2 and the MoSe_2_-*m*QDs-*L*. The PLE spectra showed that as the λ_ex_ increased, the PL peaks of MoSe_2_-*m*QDs-*S* more red-shifted in pH 7 and 11 than in pH 2. However, in MoSe_2_-*m*QDs-*L,* the red-shift of the PL peak was less than those in MoSe_2_-*m*QDs-*S*. From the TRPL results, the excitons in MoSe_2_-*m*QD dominantly decayed with an intrinsic energy bandgap in pH 2, while in pH 7and 11 they clearly migrated into the lower energy states. The PL peaks of MoSe_2_-*m*QD- showed a small red-shift compared to those shown in MoSe_2_-*m*QD-*S*. Consequently, the variation of pH is more effective on the photophysical properties of MoSe_2_-*m*QD than the size effect. We expect these findings to expand the understanding of photophysical properties of MoSe_2_-*m*QD, which will be helpful for potential applications.

## Figures and Tables

**Table 1:** Phtotophysical parameters of MoSe_2_-*m*QD.

Materials	Size (nm)	pH	Peak (nm/eV)	FWHM (eV)	QY (%)	Abs @ 325 nm
MoSe_2_	3	2	418.3	0.79	3.7	0.326
		7	427.5	0.9	5.2	0.374
		11	425.3	0.9	5	0.352
	12	2	437.6	0.84		
		7	461.3	1.07	0.4	0.689
		11	461.9	1.08		

## Data Availability

Not applicable.
